# Redirecting venous flow from the superior mesenteric vein to the inferior mesenteric vein in resections for locally advanced pancreatic cancer

**DOI:** 10.1007/s00423-025-03818-1

**Published:** 2025-08-29

**Authors:** S. K. Burgdorf, M. Pai, N. H. Habib, J. H. Storkholm

**Affiliations:** 1 Department of Digestive Diseases, Transplantation and General Surgery, Rigshospitalet, Copenhagen, Denmark; 2 Department of HPB Surgery, Imperial College, Hammersmith Hospital, London, UK

**Keywords:** Pancreas, Cancer, Surgery, Advanced venous resection

## Abstract

**Objective:**

To evaluate the outcome of ligation of the superior mesenteric vein (SMV) and redirection the venous flow through the inferior mesenteric vein (IMV) in selected patients with pancreatic cancer.

**Background:**

Radical surgery is the only potentially curable treatment of pancreatic cancer. Tumor-invasion of the SMV and its attributes will in some cases make it impossible to reconstruct the venous flow.

**Methods:**

Consecutive patients with pancreatic cancer operated between January 2019 and December 2022 were included. Selected patients eligible for venous flow reversal after systemic chemotherapy were offered surgical exploration.

**Results:**

Eleven patients were offered surgery and nine of these went through radical resection with redirection of the venous flow through the IMV. True histological R0 resection was achieved in 6 (67%) patients. No 30- nor 90-days mortality was recorded. Median overall survival was 23.2 ± 11.5 months.

**Conclusion:**

Redirecting venous flow through the IMV while sacrificing the SMV during pancreatic resection is a safe procedure and may provide the opportunity for curative resection in otherwise unresectable patients.

**Supplementary Information:**

The online version contains supplementary material available at 10.1007/s00423-025-03818-1.

## Introduction

Despite advances in modern medicine, pancreatic ductal adenocarcinoma (PDAC) continues to have a poor prognosis. More than that, the incidence is increasing. In 2020, pancreatic cancer accounted for 2.6% new cancer cases and 4.7% of cancer deaths worldwide, making it the seventh leading cause of cancer-related deaths [1]. Although diagnosis and treatment have improved over the past years, the overall 5-year survival remains roughly 10% [2]. The main difficulties in the treatment of PDAC is late detection of most cases, due to the unspecific symptoms especially for tumors located in the body and tail of the pancreatic gland, the early local spreading through perineural and venous invasion, and its rapid (metastatic) progression [3–5]. The generally poor response to oncologic treatment marks another obstacle, making surgery the only potential curative treatment. At the time of diagnosis, only about 20% of patients have surgically resectable disease [4, 6]. Along with neoadjuvant regimens, the resection of encased visceral arteries in patients with borderline or locally advanced disease is a new approach to treat otherwise palliative patients.

Resection of the superior mesenteric vein (SMV) is nowadays common practice in high volume centers and the long-term survival after radical operation for tumors invading the SMV is comparable to patients without invasion [7]. Techniques for reconstructing the mesenteric–portal axis differ according to the extent of venous resection and have been classified into four types by the International Study Group of Pancreatic Surgery (ISGPS) [8].

However, venous resection and reconstruction is not always straight forward. Involvement of first-order branches of the SMV is a surgical challenge. In a report by Katz et al. [9], segmental resection of one of the two first-order branches of the SMV could be performed without reconstruction if the remaining branch was preserved and assured collateral mesenteric venous drainage. Acute thrombosis of the PV and SMV can lead to mesenteric venous ischemia and necrosis. Thus, the lack of feasible reconstruction has historically been a contraindication of pancreatic resection.

Data from trauma literature suggests that SMV ligation can be performed without dramatic consequences [10]. In cases of chronic SMV obstruction by a tumor or previous thrombosis, development of collateral flow through the inferior mesenteric (IMV) and splenic veins (SV) allows ligation of the SMV without reconstruction. Some patients with severe distal SMV stricture/thrombosis and a favorable anatomy with an uninvolved SV receiving the IMV may be resectable with sacrifice of the distal SMV and reversal of intestinal drainage via the IMV.

We present a series of resection the SMV without reconstruction or by-pass but with intestinal venous flow reversal through the IMV or IMV/SV confluence.

## Material and methods

Between January 1 st 2019 and December 31st. 2022, 1186 patients with pancreatic tumors were operated at our institutions of these 1044 patients (88.0%) had resectable disease. 38 of these patients (3%) were identified at our MDTs (Multi Disciplinary Team Conference) to be eligible for downstaging chemotherapy due to locally advanced tumors and were due to response to chemotherapy offered exploration and possibly resection. Eleven patients with non-reconstructable SMV-involvement underwent exploration and of these nine patients were resected, resulting in flow reversal procedures (0.8%). In the same period, 142 patients (12.0%) had unresectable disease and underwent surgical exploration only, see flowchart.

Resectability was evaluated preoperatively at our MDT conferences in the presence of radiologists, interventional radiologists, nuclear physicians, oncologists, and surgeons. Diagnostic imaging included a three-phase multi-detector-row computed tomography (MDCT) and, if needed, supplementary magnetic resonance imaging (MRI) and/or positron emission tomography-computed tomography (PET-CT).

The venous vascular anatomy and involvement was evaluated using the portovenous phase of the MDCT. MRI and PET CT was used to detect signs of metastatic disease. The criteria for eligibility for attempted venous flow reversal were:

Involvement of the SMV below the SV/IMV confluence affecting the junction of the contributing major mesenteric veins necessitating more than two anastomoses.

Favorable anatomy: Well calibrated IMV with a diameter of more than 8 mm (anastomosis possible). Anatomy with a free IMV junction above the intended division of the SMV or with IMV junction with the SV with at least 10 mm distance to the tumor (anastomosis possible).

Patients with severe collateral formation were not considered eligible for flow reversal.

After the MDT conference, the surgeon and an anesthesiologist evaluated the operability of patients with resectable tumors. Preoperative risk assessment was graded according to the American Society of Anesthesiologists classification (ASA) [11]. All patients were treated with systemic chemotherapy for at least 6 months before surgical exploration.

Operation time, perioperative blood loss, postoperative hospital stays, and complications were recorded. Postoperative complication score was based on the Clavien-Dindo classification of surgical complications [12]. The International Study Group on Pancreatic Surgery (ISGPS) classification was used to score postoperative pancreatic fistula, delayed gastric emptying, and post-pancreatectomy hemorrhage [13–15]. Surgical site infection was defined according to the Center for Disease Control and Prevention (CDC) definitions [16]. Ischemic morbidity was defined as an abdominal organ complication caused by surgery-related ischemia. Perioperative death was defined as all deaths occurring during hospitalization and/or within 90 days of surgery. Events related to survival were measured from the time of surgery. Length of hospital stay was defined as time from surgery to discharge. Tumor stage was classified according to the AJCC cancer manual 8^th^ edition [17]. The resection margin status (R-status) was evaluated according to the general recommendations by the Royal College of Pathologists definition and was classified as R0 (no residual, distance margin to tumor > 1 mm), R1 (residual tumor, distance margin to tumor < 1 mm), and R2 (residual tumor, macroscopically positive margin) [18]

## Operative technique

During the operation a thorough exploration with intraoperative ultrasound was performed. All operations were performed as"artery first"procedures [19], a wide Kocher’s maneuver and opening the lesser omentum provided access to the origin of the SMA and the CA and its branches to exclude tumor invasion.

The SMV, IMV and SMA were dissected at the lower margin of the pancreas using a combined inframesocolic and left posterior approach. Biopsies were collected at the base of the mesentery. If frozen sections were without tumor cells the procedure continued. Then a complete Cattell-Braasch maneuver was performed to completely control the mesenteric root. The flow in the IMV was measured using transit time flow measurement (TTFL) whereupon a trial clamp of the SMV was performed and the IMV flow was measured again. If the flow rates increased more than 50% and there were no signs of stasis in any peripheral intestinal veins the procedure was continued.

The hepatoduodenal ligament was dissected, and the portal vein was mobilized to the hepatic hilum. This was followed by cholecystectomy, division of the common hepatic duct, the stomach, and the proximal jejunum. Depending on location of the tumor a Whipple procedure or a total pancreatoduodenectomy was performed, in which cases the spleen was also resected. This dissection left the entire specimen attached only to the area with tumor growth around the vein. The SMV clamping procedure was repeated and the flow patterns were noted. The SMV was ligated and cut and depending on the IMV anatomy a venous reconstruction was performed either as a direct anastomosis between the IMV and PV or between the SV and PV in cases where the IMV was originating from the SV and this was not tumor involved (see Table 1).

**Table 1:** Pre- and per-operatative data

Patient No	Gender	Age	BMI	ASA score	Pre Chemo CA19-9	Pre-OP CA 19–9	PreOP Chemo	Surgical procedure	Per-OP bleeding	OP time (min)
1	F	39	28	1	811	112	FOLFIRINOX	PD	450	258
2	F	71	22	2	3225	80	FOLFIRINOX	TP + PV-LGV/SV/IMV anastomosis	1100	236
3	M	68	31	2	187	211	FOLFIRINOX (r)	PD	600	195
4	M	65	27	2	265	3	Gem-NabPaclitaxel	PD + PV-SV/IMV anastomosis	400	220
5	F	49	33	1	6755	87	FOLFIRINOX	TP + PV-IMV anastomosis	1500	340
6	M	55	20	1	318	19	FOLFIRINOX (r)	PD	650	310
7	F	60	25	2	2213	7	Gem-NabPaclitaxel	TP + PV-IMV anastomosis	750	210
8	F	58	22	1	98	183	FOLFIRINOX (r)	TP + PV-LGV/SV/IMV anastomosis	900	280
9	M	56	25	3	2093	139	FOLFIRINOX	PD	2000	336

Extended lymphadenectomy including stations 8p, 12a, b, p and 16b1 was routinely performed.

**Fig. 1:**
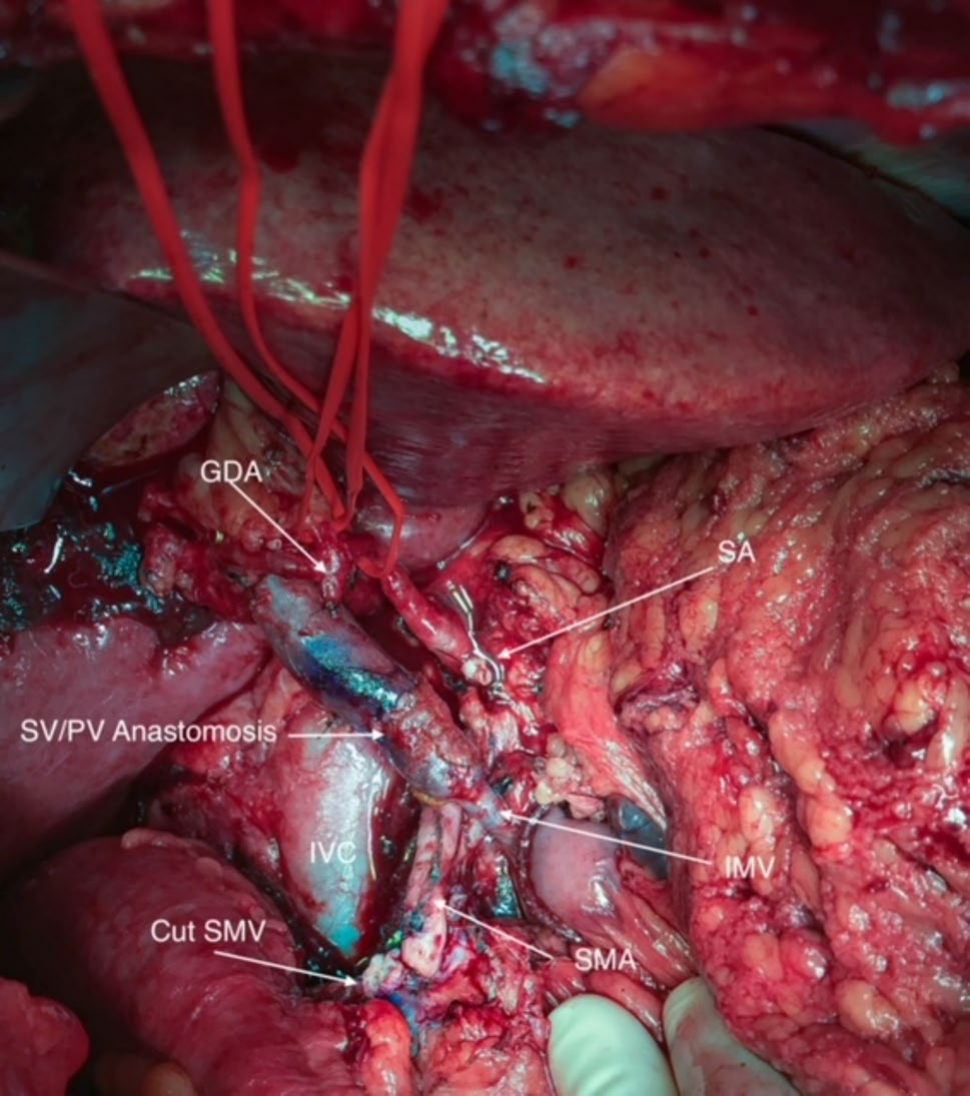
Final operative field after PD with resection of SMV and venous flow reversal through IMV/PV. GDA: gastroduodenal artery, SA: splenic artery, SV: splenic vein, PV: portal vein, IMV: inframesenteric vein, SMA: supramesenteric artery, SMV: supramesenteric artery

The remaining steps of the procedure followed a standard Whipple/total pancreatectomy procedure with pancreatico-jejunostomy, hepatico-jejunostomy, and gastro-jejunostomy. For final operative field please see Fig. 1.

Thrombosis prophylaxis with LMW heparin, Klexane® 40 mg q.d was given until 28 days after surgical procedure. Patients were followed daily the first three postoperative days with bedside doppler ultrasonography to verify venous flow.

## Results

This study included nine patients all with locally advanced PDAC involving most first order SMV contributories. All patients received preoperative downstaging chemotherapy with four of them receiving full dose FOLFIRINOX and three receiving reduced dose FOLFIRINOX regimen for 6 months. Two patients received Gemcitabine and Nab Paclitaxel for six months. Seven patients received adjuvant therapy (Four FOLFIRINOX and two Gemcitabine monotherapy). To patients opted out of adjuvant chemotherapy.

Histological examination of the specimens showed invariably ductal adenocarcinoma.

Six patients (67%) had R0 resections and three (33%) patients R1 resections. No patient had tumor invasion at the radial, mesenterial or posterior margins. All patients had N disease and two patients were classified as M1 because of metastatic adenocarcinoma in Station 16b1.

Three patients had standard PD with sacrifice of the SMV below the splenic vein and did not need any vascular anastomoses.

One patient had severe collateral formation and required an intraoperative temporary bypass to the portal vein using a PTFE graft.

The average hospital stay was 17 days.

One patient had a grade B pancreatic fistula treated with external drainage. One patient developed chylous ascites and was treated with low MCT diet for one week. A patient had DVT and small peripheral pulmonary embolisms and was treated with anticoagulation therapy for three months. Postoperative routine doppler ultrasound demonstrated normal portal venous flow in all patients. On oncological routine CT scans no patients developed thrombosis in the altered intestinal venous drainage.

There was no 30- nor 90-day mortality.

Median survival was 23.2 ± 11.5 months. Three patients have died, and the remaining patients are still alive. The three patients were not diagnosed with recurrent disease during routine oncological surveillance, which included CT imaging and CA 19–9 measurements every three months One patient developed liver metastases 30 months after surgery and another patient had an isolated local recurrence 9 months postoperatively. Both patients are still alive (see Table 2).

**Table 2:** Postoperative data

Patient No	Hospital stay (days)	Complications	AJCC stage	R status	Adjuvant Chemotherapy	Recurrence	Status Dead/Alive
1	15	DVT and peripheral pulmonary embolism	ypT3N2M0	R1	Gemcitabine	local recurrence 9 months	Alive 10 Months
2	10	none	ypT2N1M0	R0	no	-	Dead 12 months
3	7	none	ypT3N2M1*	R0	FOLFIRINOX (r)	-	Dead 29 months
4	18	Chylous ascites	ypT3N2M0	R1	FOLFIRINOX	Liver met 30 months	Alive 38 months
5	25	fistula hepeticojejunostomy + PTC	ypT1N2M0	R0	FOLFIRINOX	no	Alive 33 Months
6	13	Grade B fistula	ypT3N2M0	R1	Gemcitabine	-	Dead 36 months
7	11	none	ypT2N2M1*	R0	N	no	Alive 8 months
8	9	none	ypT3N1M0	R0	FOLFIRINOX (r)	no	Alive 20 months
9	42	Intra-abdominal collection (drain), re-laprotomy for closure of wound	ypT2N0M0	R0	FOLFIRINOX	no	alive 23 months

## Discussion

Patients with PDAC involving the PV/SMV are potentially curable with a combination of systemic chemotherapy and radical surgical resection. True surgical radicality can be challenging depending on the location and extend of tumor invasion of the veins. Morbidity and rates of portal vein thrombosis are increased with higher complexity of the portal vein resection, as defined by the ISGPS [8, 20].

In cases of PDAC with involvement of first-order branches of the SMV the difficulty of venous reconstruction and vascular complications increases [9]. Many studies have not found any significant differences in terms of postoperative morbidity and mortality after venous resection [21, 22]. Other authors have previously relied on preserving massive collaterals when resecting the SMV without reconstruction [23]. This technique is obviously highly dependent on well-established venous collateral flow in venous plexuses that can be spared when resecting the specimen. Perioperative bleeding in these thin walled dilated venous plexuses can be a technical challenge, especially when knowing that the vital venous drainage in particular from the small bowel and sufficient portovenous flow is dependent on these collateral venous networks. In our study cohort we only found one patient with pronounced collateral formation. We could accordingly not rely on collaterals for drainage to the portal system. However, in cases with collateralization careful attention to hemostasis is imperative and shunting the SMV system to the PV or the inferior vena cava may in many cases be required in order to avoid excessive blood loos. Preservation of the veins draining into the IMV is crucial to maintaining adequate mesenteric venous drainage when the occluded SMV and surrounding collaterals are resected en-bloc with the specimen.

This is, to our knowledge, the first series specifically establishing an alternate non collateral venous drainage of the gut via the IMV system to the PV in patients with tumors invading the confluences of the mesenteric veins. These patients would in many centers be considered unresectable.

With a median overall survival of 23.3 months, these selected patients have achieved overall survival rates comparable to up-front resectable patients with venous involvement [20]. The only alternative for most of these patients would have been palliative oncological treatment with a dismal prognosis.

Long-term results after PV reconstruction have shown 1- and 5-year patency rates of 50%­80% and 17%, respectively [24]. There have been some cases of early thrombosis after PV reconstruction, with no major complications. Stenosis of the PV anastomosis area has been shown to be associated with local recurrence [25–28].

A recent study from the high-volume pancreatic cancer center in Heidelberg with almost 700 cases of venous resection compared to more than 1500 cases without venous resection have shown that morbidity, and in these cases especially portal vein thrombosis, increases with higher venous resection type (ISGPS). The rate of portal vein thrombosis for patients going through pancreatic surgery with any type of venous resection was 7.2%, whereas the thrombosis-rate was three times higher for patients with graft interposition (type 4, ISGPS) 21.2%. There was no significant difference in 30- and 90-day mortality between the groups [20].

In most cases requiring venous reconstruction, end-to-end anastomosis is possible. If the distance between the veins are long and mobilizing the liver, dissecting the PV completely to the confluence and doing Cattell-Braasch maneuver is not enough to do a direct end-to-end venous anastomosis, some authors have described cases with the use of autologous venous grafts or prosthetic patches with acceptable postoperative outcomes [7].

While SMV resection without reconstruction and flow reversal is a novel concept, there are several studies evaluating postoperative stenosis or thrombosis of venous anastomoses with patency rates at one year of up to 80% [21, 22]. It is, therefore, interesting that we did not observe stenosis or thromboses in any patient. This is most likely a consequence of sufficient flow and minimal turbulence in the established alternative route.

In this series we completely sacrificed the SMV and any collaterals around this vessel. This meant that we could be very aggressive in the resection of the tumor allowing for a wider resection margin in the retroperitoneum. This may explain the relatively high number (63%) of true R0 resections.

We had no 30- or 90-days mortality. Median survival of 23.3 months is comparable to other studies of complex venous involvement [7, 9]. However, as our cohort comprises only nine patients, it is difficult to draw any solid conclusions regarding long term survival.

## Conclusion

Venous flow reversal via the IMV seems to be a safe procedure in carefully selected patients. Furthermore, flow reversal may be useful in patients with otherwise unresectable pancreatic tumors at the lower margin of the pancreatic head and uncinate process that involve most first order mesenteric veins necessitating sacrifice of the SMV to achieve an R0 resection.

This study clearly has limitations: This is a highly selected patient population with a N of 9. The procedure seems feasible and safe. However, further high-volume multicenter studies are needed to achieve a significant number of patients to be able to draw conclusions on the impact on long term survival.

## Supplementary Information

Below is the link to the electronic supplementary material.

Supplementary file1 (DOCX 25 KB)

## Data Availability

No datasets were generated or analysed during the current study.
