# Supplementary material for: Redirecting venous flow from the superior mesenteric vein to the inferior mesenteric vein in resections for locally advanced pancreatic cancer
Source: Langenbecks Arch Surg. 2025 Aug 29;410(1):257. doi: 10.1007/s00423-025-03818-1 (PMC12397166; doi:10.1007/s00423-025-03818-1)
Supplement: Supplementary file 1 — Supplementary file1 (DOCX 25 KB) [file 423_2025_3818_MOESM1_ESM.docx]

Flowchart

**9 pts resectable with flow reversal procedures**

2 pts non-resectable at exploration

11 pts non-reconstructable SMV-involvement

1186 pts operated

1006 pts up-front resectable

142 pts non-resectable at exploration

38 pts downstaging chemotherapy
